# Investigating Additive Manufacturing Processes of Polymeric Materials with X-ray Scattering Techniques

**DOI:** 10.1063/4.0001006

**Published:** 2025-10-27

**Authors:** Lutz Wiegart

**Affiliations:** National Synchrotron Light Source II, Brookhaven National Laboratory, Upton, NY, USA

## Abstract

Advanced manufacturing processes, such as 3D printing of polymeric materials, often involve transitions of the materials from a complex (non- Newtonian) fluid to a solid state. These out-of-equilibrium processes follow a complex energy landscape, resulting in spatial and temporal heterogeneities that ultimately determine the final structure, functionality, and defects of the materials. In order to successfully 3D print polymer nanocomposites with designed sub-filament mesoscale structures, it is crucial to have a thorough understanding and control over the various processes that occur during both the printing and post-processing stages. To achieve this, we have developed a combination of in-situ instrumentation and synchrotron-based time-resolved (coherent) microbeam X-ray scattering techniques. These techniques allow us to accurately measure material dynamics, structure, strain, and defect formation, providing insight into the mechanisms behind structure formation at relevant spatial and temporal scales in polymer nanocomposites.

This presentation will discuss examples of 3D printing processes that have been studied in-situ using time-resolved coherent small angle scattering, including pre-ceramic inks, thermosets, and dual-cure (UV/thermal) epoxy nanocomposites.

